# Leveraging innovation, education, and technology for prevention and health equity: Proceedings from the cardiology oncology innovation ThinkTank 2021

**DOI:** 10.3389/fcvm.2022.982021

**Published:** 2022-09-29

**Authors:** Sherry-Ann Brown, Generika Berman, Jim Logan, Diego Sadler, Rohit Moudgil, Brijesh Patel, Marielle Scherrer-Crosbie, Daniel Addison, Richard K. Cheng, Arco J. Teske

**Affiliations:** ^1^Cardio-Oncology Program, Division of Cardiovascular Medicine, Medical College of Wisconsin, Milwaukee, WI, United States; ^2^Medical College of Wisconsin, Green Bay, WI, United States; ^3^University of Wisconsin-Milwaukee, Milwaukee, WI, United States; ^4^Cardio-Oncology Section, Department of Cardiovascular Medicine, Heart, Vascular and Thoracic Institute, Cleveland Clinic Florida, Weston, FL, United States; ^5^Section of Clinical Cardiology, Department of Cardiovascular Medicine, Heart, Vascular and Thoracic Institute, Cleveland Clinic, Cleveland, OH, United States; ^6^Section of Cardiology, Department of Medicine, West Virginia University, Morgantown, WV, United States; ^7^Division of Cardiology, Department of Medicine, Hospital of the University of Pennsylvania, Philadelphia, PA, United States; ^8^Cardio-Oncology Program, Division of Cardiology, The Ohio State University Medical Center, Columbus, OH, United States; ^9^Division of Cardiology, Department of Medicine, University of Washington, Seattle, WA, United States; ^10^Division of Heart and Lungs, Department of Cardiology, University Medical Center Utrecht, Utrecht, Netherlands

**Keywords:** innovation, prevention, health equity, cardiology, oncology, artificial intelligence, digital health, digital transformation

## Introduction

Cardio-oncology has emerged as a distinct cardiology subspecialty over the last decade. While many still consider this field to be limited to anthracyclines and heart failure, cardio-oncology has expanded to include the entire spectrum of cardiology (ranging from rhythm disturbances to vascular toxicities) regarding adverse effects of cancer therapies. Modern cancer therapies continuously produce an uncharted territory of cardiovascular toxicities, such as recent developments and insights in cardiotoxicity associated with immune checkpoint inhibition. Thus, cardiologists, oncologist, and other specialists and consultants have come together to pursue innovation together in the Cardiology Oncology Innovation Network (COIN).

A range of specialists in the network gathered in August 2021 for the first ever COIN ThinkTank. The agenda items for the inaugural COIN ThinkTank 2021 meeting were determined by network members at previous COIN gatherings. The primary objective of the ThinkTank was to investigate collaborative knowledge gaps and provide a platform to facilitate the development and implementation of various forms of innovation and cross-platform communication. Our goal was to contribute to an international initiative that will propel cardio-oncology forward into the era of digital transformation and health equity. Each topic discussed at the ThinkTank was therefore considered in the context of exploring prevention, addressing inequity, and strengthening the involvement of oncologists in cardio-oncology.

The following were the predetermined topics that anchored discussions in breakout rooms during the ThinkTank:

- Artificial intelligence (AI) and digital health in cardio-oncology,
- The role of informatics in the global cardio-oncology registry (G-COR),
- Education on innovation and development of innovative educational techniques in cardio-oncology.

**Figure 1:**
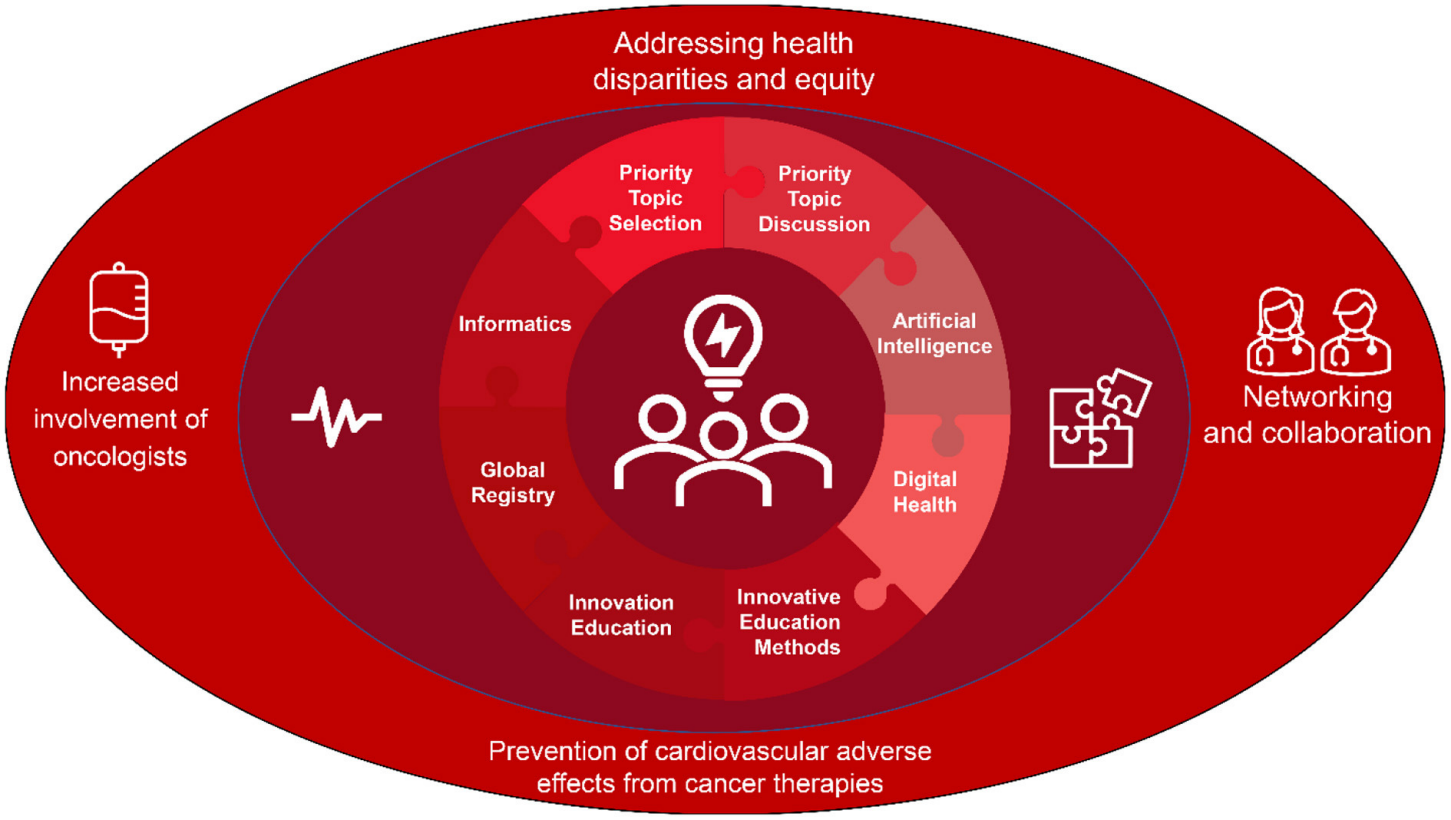
ThinkTank priority topics selected and discussed. The Cardiology Oncology Innovation Network ThinkTank 2021 focused on priority topics preselected by network members and leaders at preceding gatherings, such as the virtual receptions at the end of daytime sessions of national or international cardiology, oncology, or cardio-oncology meetings. The priority topics selected and discussed were artificial intelligence and digital health, the role of informatics in the collaborative global cardio-oncology registry, and education on innovation coupled with innovative methods of education in cardio-oncology. These priority topics were considered in the setting of prevention of cardiovascular adverse effects from cancer therapies, addressing health disparities and equity, and increasing the presence and involvement of hematologists/oncologists in collaborations in cardio-oncology.

Specific emphasis was placed on how to advance prevention efforts, eliminate racial and ethnic disparities, and increase collaboration among cardiologists and oncologists in open discussion with ample room for ideas and innovations (Figure 1). In addition to these predetermined discussion topics, participants provided additional input on future ThinkTank items, including translational research and building a consortium.

Here, we introduce the format, content, and participants of the ThinkTank, and we summarize these discussions and their implications for the future of cardio-oncology in the context of the tripartite COIN mission: innovation, collaboration, and education.

## Format and structure of ThinkTank

The 2021 COIN ThinkTank was held on August 7, 2021 and was designed to facilitate meaningful discussion and the dissemination of information. An interactive meeting on Zoom was convened for 3 h. Dr. Sherry-Ann Brown welcomed the attendees and provided an introduction. The majority of the remaining time was devoted to three 40-min breakout room sessions, separated by 5-min virtual exhibit engagement breaks and room transfers. Small working groups discussed the following topics in the context of prevention and disparities and increasing involvement of colleagues in oncology: AI and digital health in cardio-oncology (innovation); the role of informatics in G-COR hosted in Research Electronic Data Capture (REDCap) Cloud (collaboration); education on innovation and innovative delivery methods for education in cardio-oncology (education).

After introductions in the main room, the breakout rooms were opened. Then individuals who were not serving as breakout room leaders selected their desired destination breakout rooms. Three breakout rooms each lasting for 40 min were facilitated, in order to offer discussants the opportunity to rotate among all three breakout rooms before the end of the ThinkTank. Consequently, breakout room leaders remained within their designated breakout rooms while participants rotated among the rooms as desired. This allowed the accumulation of summary points while new ideas were incorporated by different individuals in each room. The breakout room leader roles included a group facilitator, scribe, timekeeper and technical support. The group facilitator led the group discussions. The scribe took notes and reported out for the group toward the end of the ThinkTank. The timekeeper let the group facilitator know when 40 min passed during discussions. Finally, the individual in the technical support role checked in with the main room and breakout room to ensure all participants were in rooms as desired.

The individuals in breakout room 1 focused on AI and digital health to answer the following questions.

- How can we collaborate on AI and digital health to advance prevention efforts in cardiology, oncology, and cardio-oncology?
- How can we collaborate on AI and digital health to advance efforts at eliminating ethnic and racial disparities in cardiology, oncology, and cardio-oncology?
- How can we increase collaborations among cardiologists and oncologists on topics relevant to AI and digital health?

The participants in breakout room 2 focused on the role of informatics in G-COR sought to answer the questions:

- How can we collaborate on the role of informatics in G-COR to advance prevention efforts in cardiology, oncology, and cardio-oncology?
- How can we collaborate on the role of informatics in G-COR to advance efforts at eliminating ethnic and racial disparities in cardiology, oncology, and cardio-oncology?
- How can we increase collaborations among cardiologists and oncologists on topics relevant to the role of informatics in G-COR?

The attendees in breakout room 3 focused on education on innovation and innovative delivery methods for education in cardio-oncology sought to answer the following questions.

- How can we collaborate on education on innovation and innovative delivery methods for education to advance prevention efforts in cardiology, oncology, and cardio-oncology?
- How can we collaborate on education on innovation and innovative delivery methods for education to advance efforts at eliminating ethnic/racial disparities in cardiology, oncology, and cardio-oncology?
- How can we increase collaborations among cardiologists and oncologists on topics relevant to education on innovation and innovative delivery methods for education?

Following the breakout room discussions, each group was designated 5 min to report out what had been discussed in the small groups. Then cardio-oncology poetry and closing remarks were shared to conclude the event.

## Expert participants

Breakout room participants were cardiology, oncology, and industry leaders, as well as patient advocates spanning geographic locations and institutions from across the United States and the world. Attendees had a range of interests within cardio-oncology, as well as diversity of career and training stages. Here we provide excerpts from some of our participants' introductions to illustrate the spectrum of expertise.

A specialist with a dual role in cardio-oncology and primary care at the Royal Brompton Hospital in London described frequently following patients from primary care to oncology to cardio-oncology and back to primary care. Thus, she sees the patient's entire treatment cycle, which she finds fascinating. She mentioned her interest in AI and digital technology, as evidenced by the availability of her presentation on the fundamentals of AI hosted online by the European Society of Cardiology. With Microsoft and Hitachi, her group is attempting to create a prototype cardio-oncology work management system in England. Their goal is to automate many of their processes, which currently require substantial human input, in order to increase efficiency and throughput.

The chief executive and innovation officer of a digital health company co-founded with the assistance of several doctors, engineers, and financial professionals also described her work with an insurance company, concentrated on elder care and the management of chronic conditions, with a focus on healthcare finance payments. Their projects on AI in healthcare, and prior with IBM Watson, were also discussed, guided by a patient-centered approach. As a previous nurse case manager, she described her clinical background, and her current natural aptitude for technology, as she delves into quantum computing.

A faculty member at the University of Pennsylvania in Philadelphia described her work with multiple cardio-oncology trials utilizing echocardiography and more recently cardiac MRI. She described her experience with the role of AI in cardiac imaging and cardio-oncology, contributing to a very fruitful discussion.

Our patient advocate discussed his work with creating digital media, producing webcasts, and a variety of other tasks, such as video production. He described his status as a cardio-oncology patient, having participated in the very first imatinib clinical trials and is now no longer on cancer therapies.

A director of program grants and strategic partnerships at an academic institution was also present. He reported learning a great deal in the AI breakout room and described his professional role locating funding sources for the cancer center's work.

A bioengineering lecturer at Santa Clara University had previously spent many years as an engineer and executive typically working with cardiology devices. Consequently, medical device development was her area of expertise. She also provides consulting services to medical technology startup companies. She described contact with the COIN founder, who had given a guest lecture on cardio-oncology, which inspired her greatly. Therefore, she joined the network and participated in the ThinkTank to listen in this space and determine the unmet needs of physicians and patients that may benefit from her background.

A cardiologist and echocardiographer from the National Institute of Cardiology in Mexico City described being extremely focused on the notion that precision medicine and AI are the means by which we can treat more cardiovascular disease and cancer patients. He discussed involvement of members of the Mexican Society of Cardiology in the COIN ThinkTank, along with colleagues attending from Colombia and Argentina and others from South America.

A pediatric cardiologist, echocardiography specialist, and bariatric interventionalist from Mexico City who focuses on the evaluation of children undergoing cancer treatment and subsequent childhood cancer survivors described working to disseminate information from related fields, beginning with raising awareness of the issue of cardio-oncology in Mexico.

All of these experts and several others gathered to discuss these innovative topics and how to navigate them together in cardio-oncology.

## Key insights and professional guidance

### Innovation

#### Advances in artificial intelligence and digital health

In parallel, progress has occurred in oncology, cardiology, and other fields invested in AI and digital transformation. We can learn from these accomplishments to understand innovations in AI. We can apply these discoveries to the interrogation of existing databases such as (Surveillance, Epidemiology, and End Results) SEER and Medicare, in collaboration with each other and with statisticians. Appropriate resources and funding in the form of National Institutes of Health (NIH) and institutional grants and beyond are needed to facilitate these collaborations providing valuable information to drive innovation forward in cardio-oncology.

AI could be applied to the development of a curated repository for cardio-oncology imaging, to include tests such as MRI, echocardiography (including at the point-of-care), CT, ECG, and PET/CT. These imaging tests are frequently obtained for patients as part of their cancer surveillance and staging. Cardiologists can partner with oncologists and radiologists to streamline the gathering of these studies for AI work in cardio-oncology. It is imperative that we work together to channel these potential opportunities and maximize opportunities for AI. These efforts requires substantial work, time, and effort, and the results are worth it. Interrogation of existing databases, such as Medicare and SEER, can be challenging. AI may help simplify and interpret some of this output. A statistician can also be key in these studies. These are all reasonable opportunities to pursue. Based on results, an app can be created in the future to incorporate additional validation studies.

Individuals from cardiology, oncology, and other specialty areas participate in COIN. As various areas of medicine, healthcare, and industry have already begun using AI, we can leverage this pre-existing expertise and develop creative solutions. We can explore pre-existing AI work and learn from them and invite various experts in AI to serve as mentors as we collectively pursue these studies funded by governmental and non-governmental organizations.

In the future, even beyond AI alone, we will also incorporate efficiency through quantum computing, as well as remote health monitoring, telehealth, and digital health. We anticipate ongoing partnership with companies and computer programming and biomedical engineering programs that develop software as well as the hardware. Therefore, we will have the ability to co-create and add components, whether to watches with ECG capabilities, or to other wearable devices with a variety of medical tools.

#### Present and future artificial intelligence applications

AI applied to cardiac MRI can be used to obtain information regarding myocardial structure and mapping to gain insight into global and regional heart function and muscle contractility. These can give insight into tissue composition, which can be coupled with more advanced information about metabolomics and other -omics in precision imaging. By using MRI for early markers, separating patients based on treatment/cancer type, and utilizing AI algorithms to calculate strain and ejection fraction, the cardio-oncology clinic can be transformed. What training is required and what is the potential of this technology? What will the future hold?

It is possible to have individuals without echocardiography training perform echocardiograms, augmented by AI in real-time. AI guides the person obtaining the echocardiogram to obtain the appropriate images and can automatically calculate the ejection fraction. This work is also being pursued for automatic point-of-care assessment of strain. In such ways, AI might be able to transform the cardio-oncology clinic. As echocardiographers in cardio-oncology, of course we maintain that individuals generally should be trained formally in echocardiography. Nevertheless, where this is not readily available, point-of-care AI-guided echocardiography may become key.

Some companies are currently researching myocardial strain for early detection of subclinical myocardial dysfunction, to improve options for disease prevention. As a result, we are attempting to determine the best parameters for predicting post-treatment ejection fraction decline. AI can be used in this way, applied to cardiac imaging, as well as to various studies examining the effects of radiation and chemotherapy, and other forms of cancer treatment for breast, lung, and various cancers on the cardiovascular system.

Future applications of AI and digital health innovation in cardio-oncology will also involve using the Substitutable Medical Applications and Reusable Technologies (SMART) on Fast Healthcare Interoperability Resources (FHIR) architecture to incorporate apps into Epic *via* the Epic App Orchard, working in and with virtual reality, and working routinely with data scientists, multidisciplinary scholars, on various research efforts. Innovative methods for file sharing and data storage will be needed, in addition to applications for large multi-institutional grants to kickstart funding. Working with Nvidia's GTX which has quantum computing capabilities should be considered.

### Collaboration

#### Informatics in G-COR

The global cardio-oncology registry (G-COR) is a recently launched multi-center, global registry (1). Informatics can play a variety of roles in G-COR, including data abstraction and curation, risk calculator generation and output, and tracking the evolution of cardiovascular health of participants in the registry. G-COR is based in over 20 countries and has over 120 academic and community centers as participants. Prospective collection of clinical data from patients with breast cancer has begun, in the pilot phase of the program. Subsequent stages will incorporate data for patients with hematological malignancies and those treated with immune checkpoint inhibitors for various cancers. This global registry will generate vast quantities of clinical data, providing a unique opportunity to place a strong emphasis on disparities in access to cardio-oncology, barriers to care access, and regional disparities. New developments in automated data extraction from electronic health care systems (e.g., Epic) will be investigated to further facilitate the entry of accurate and comprehensive data into this registry. Leveraging data for transformation in cardio-oncology will be a focus for the using informatics in the registry.

The ThinkTank identified two additional potential advantageous benefits of applying informatics in G-COR: (1) assessing valuable feedback from the participating centers, which are typically led by experienced cardio-oncologists; and (2) providing feedback to these centers, allowing them to compare their numbers and data to the global registry in order to assess their own strengths and weaknesses. The latter would be anticipated to have a direct effect on local healthcare policies and protocols.

#### Facilitating prevention through collaboration in G-COR

Preventive informatics can be advanced in G-COR, with the development of robust and risk calculators validated in prospective cohorts in the registry. In addition, practice variations in different centers, regions, and countries should be studied. By analyzing the different approaches, it would be possible to identify the most effective pathways for preventing cardiovascular toxicity.

Advanced informatics and analytics algorithms are needed to facilitate cardio-oncology referral patterns for patients at highest risk of cardiovascular disease, in order to facilitate preventive efforts. Informatics can be used to identify cancer survivors who are being undertreated for their cardiovascular risk or disease. Informatics projects can be devised to intervene in these cases and incorporate AI and predictive analytics into the electronic health record.

For cardio-oncology programs in the United States, some groups may be able to study social determinants of health such as zip codes (see https://www.ahrq.gov/sdoh/data-analytics/sdoh-data.html) and access to cardiologists/cardio-oncologists, regarding their association with and ability to predict cardiovascular toxicities. Patients can potentially be supported to enter their own information such as social determinants of health, as well as adverse events (see https://healthcaredelivery.cancer.gov/pro-ctcae), if this can be pursued securely and with informed consent. State-level social determinants of health information can be collected without consent, but granularity is sacrificed. However, G-COR zip codes will not be available for collection or analysis since G-COR will not be collecting identifiable patient information.

A consideration of bias might arise if we automate Epic data abstraction, i.e., the G-COR cohort could become skewed toward larger academic centers readily able to pursue this automation and provide large amounts of real-world data. This concern will need to be addressed by supporting data gathering from smaller centers. This would also be the case for Epic-based algorithms for modeling outcomes to target for prediction and prevention based on socioeconomic factors, as data needs to be captured especially from centers with patient populations that are underrepresented. In many cardio-oncology centers, referrals are routed through patient/nurse navigators. These navigators can be trained in Epic predictive analytics and patient advocates from underrepresented groups can help guide these informatics efforts to maximize their impact for these populations.

### Education

#### Patient and clinician partnership in education on innovation

It is important to educate both doctors and patients as partners in innovation. Patient and clinicians can learn from each other and become better informed by engaging in meaningful conversation. For patients, the focus is on information that educates and empowers them to be informed participants in their health care. Thus, health education materials should be designed to reflect the context of patients' lives, explain the inter-relationships between choices and offer practical approaches to making those choices. Additionally, getting input from patient advocates can be very informative for educating clinicians in cardio-oncology. Consequently, we engage patient advocates in our network for bidirectional support and education.

#### Innovative delivery methods for education

##### Patient journey map

As we innovate for our patients, it may be helpful for us to map out the patient journey, beginning with the initial cancer diagnosis and including touchpoints with family, friends, physicians, nurses, and other health care professionals. Fears can be addressed along the journey, such as limited awareness about cancer radiation and drugs, particularly regarding assessment and comprehension of cardiovascular risk vs. benefit. An example patient journey map is one created for chronic obstructive pulmonary disease (COPD) based on social listening (2).

Sometimes patients develop frustration from not knowing or understanding their disease or risk, and express fear about the unexpected. Mapping out the patient journey and touchpoints for disease or risk assessment and management could be helpful in cardio-oncology and could be hosted collaboratively on the COIN website. Such a map may help with educating each other and others, to help us better understand the patient journey. As we share a map draft with colleagues and with patient advocates, we can offer others the opportunity to chime in and identify additional touchpoints. The patient journey may need to vary by country or region. Nevertheless, a conceptual detailed map can assist physicians, researchers, trainees, and entrepreneurs in identifying patients' touchpoints along the journey, with opportunity for education and intervention, as well as innovation.

The COPD map was developed based on social listening, or observations from social media (2). Thus, social media and other opportunities for listening in on patient needs and frustrations can be very helpful for building the conceptual map of the patient journey. Observing which websites patients visit and where else they search for information and what they seek to learn can help us understand the patient needs and journey. This information can be helpful for us all to understand and consider how to address the unmet needs of our patients. This can also be helpful for entrepreneurs to consider how to support physicians as we meet patient needs in this journey. New innovation and technologies that would aid this patient journey could be devised collaboratively, in academia-industry partnerships (3).

##### Digital collaboration

An education emphasis working group has been established on the COIN website to provide space for the group to connect and collaborate on ideas such as the patient journey concept map and collaborate together. Output from the group discussions can be posted on social media for others to view, discuss, and come up with additional ideas. This would serve as a great guide for much of our work in the network.

##### Infographics

It is important it is to engage all patients. Infographics can facilitate this for some patients. This could be simplified with the creation of a digital plan-similar to a drawing or painting-which can serve as a map to chart a course for raising awareness among patients, patient groups, cardiologists, oncologists, and other partners in this work. Many great patient-facing infographics are, for example, available on www.cardiosmart.org providing an excellent approach to patient education. CardioSmart presents the infographics in the setting of a collection of basics about the diseases, along with frequently asked questions and also resources.

##### Knowledge dissemination

We encourage early exposure to cardio-oncology in health professional training environments. Social media efforts can help globalize this effort. These methods can include disseminating brief 10–15 min interview sessions with a host and a single presenter on the latest breakthroughs on innovation in cardio-oncology, incorporating patient advocacy on various topics. These videos would also be displayed on our centralized website (cardioonccoin.org). The links to the videos would be placed on social media for both patients and clinicians. This will supplement COIN continuing medical education (CME) in the future facilitated by stable funding sources. It is essential for patients, physicians, and educational institutions to identify champions for this work. A group effort will be needed to determine who these champions are and provide the tools to make this happen.

##### Patient videos

Physicians should display customized educational videos while patients are in waiting rooms. This would enhance the patient's awareness of cardio-oncology and complement what they gain during their time directly spent with physicians. In these videos, it would be beneficial for patients to hear patients' dialogue with cardio-oncology doctors and patients' sharing information about their journeys. Such videos could become extremely valuable.

## Special topics

### Engagement with oncology

COIN draws on experiences of collaboration between cardiologists and oncologists at the local, state, national, and international levels with a goal to further increase these collaborations. In Florida especially, these collaborations are between the American College of Cardiology and the American Society for Clinical Oncology local chapters. Similar collaborations are being forged in cardio-oncology in Illinois and California, and throughout the country and world. Colleagues in both academic and non-academic centers are engaged, across professional societies within cardiology and oncology, to advance innovation and education. More cardiologists than oncologists have traditionally been involved, in large part due to cardiologists noting the adverse effects and tracking these back to cancer therapy. Thus, the desire for more collaboration with oncologists has been borne out of these clinical and research observations. Indeed, we need more oncologists to join us. Oncology involvement varies by institution and we hope to expand this throughout the network.

The ThinkTank examined next steps for enhancing collaboration between cardiologists and oncologists. Since the majority of cardio-oncology programs are administered by cardiologists, the current strategy is to implement practice changes within the cardiology community. It will be essential to engage more oncology colleagues in both academic and non-academic hospitals to have a significant impact on cardio-oncological outcomes. One way to accomplish this is to elucidate the significance of cardiovascular care, which extends beyond survival and falls within our shared mission to provide the best long-term care for our patients.

### Eliminating racial disparities in G-COR

An objective was to investigate the potential for advancing effectors to eliminate racial and ethnic disparities. One of the primary emphases of the aforementioned G-COR is to investigate how cardio-oncology patients are treated in various regions of the world. This also includes investigating the factors that influence or restrict access to care, such as socioeconomic status, race and ethnicity, access to insurance, transportation, and internet access. It would be beneficial to learn how these various socioeconomic groups, ethnic groups, and geographic locations affect cardio-oncology care. Collaboration and the collection of massive amounts of data would be required to draw meaningful conclusions. According to the ThinkTank, this is something to strive for in order to influence policymaking and reduce existing inequalities and disparities.

It is also crucial to achieve racial and ethnic health equity by eradicating barriers to care and eradicating health disparities. This is one of our key concepts and focus areas in G-COR. How do we gather data in order to study disparities in cardio-oncology? Across the globe, how do we recognize and address health equity in cardio-oncology patients? What factors impact or limit access to care, including geographical, socioeconomic, racial, and ethnic factors? In what ways has global cardio-oncology care benefited this population? How have these patterns been affected by the pandemic? In order to answer these questions, we will investigate ethnicity, income, insurance, transportation, and internet access, as well as how different socioeconomic groups, ethnic groups, and geographic locations impact access to care. We hope to generate sufficient data to analyze and chart a course to have an impact. We anticipate contributing to policymaking for the reduction of inequalities and disparities. We will additionally attempt to accomplish these in our individual cardio-oncology environments.

### Creative expression and humanism

A cardio-oncology poem capturing the experience of the patient was then shared prior to concluding comments. As part of processing thoughts in medicine and science, some of us write poetry about the things we ponder. Some of the poems are composed after we visit with patients. Even if many of us may not directly share our individual patients' experiences, we can empathize and capture their story. The poems we therefore create can literally describe our frame of reference regarding how we experience our patients' journeys. These poems can be typically straightforward and simple to comprehend. In this case, we interpret a particular patient's journey as analogous to juggling four balls at once. The poem “Juggling Four Balls in the Air” (4) was therefore shared to illustrate this principle and ground us as we closed out the ThinkTank.

Juggling Four Balls in the Air
*It's not enough*
*That I am living*
*In the middle of*
*A pandemic*.
*Nor is it enough*
*That I was diagnosed*
*With one type of*
*Cancer*
*And I have been*
*Battling that still*.
*No*,
*Because*
*When it rains*,
*It pours*.
*It's not enough*
*That a second cancer*
*Has now been found*
*In me*.
*My body's pictures*
*Lit up like a holiday tree*.
*Nor is it enough*
*That I will need*
*Two different kinds*
*Of cancer medications*
*And also radiation*.
*Is it enough then*
*That now also*
*I have been found*
*To have a heart problem*
*As well*,
*Before I even*
*Start cancer therapy?*
*Shaking my head*.
*Juggling four balls in the air*.
*Only because*
*I have seen it done*,
*I know that I also can*.
*Shall I waltz into this*
*Nonchalantly*,
*And hope for the best?*
*Shall I bring*
*A little bit of resolve?*
*No*,
*I am not going to bring*
*A teacup of aspiration*.
*No*,
*Because*
*When it rains*,
*It pours*.
*I am going to bring*
*An avalanche*
*Of hope*,
*Resolve*,
*Dreams*,
*Faith*,
*Trust*,
*Optimism*,
*Ambition*,
*Aspiration*,
*And purpose*.
*Yes*,
*Because*
*When it rains*,
*It pours*.
*I will bring*
*A hurricane*,
*A cyclone*,
*A typhoon*,
*A tsunami*.
*These are the four balls*
*I will juggle in the air*.

The poem was inspired by the patient's attitude and strength in facing all of these challenges. Our patients continue to inspire us and motivate us in everything we do. As a result, they remind us of why we are doing what we are doing and what we need to do to assist them.

## Discussion

The Cardiology Oncology Innovation Network (COIN) has gained momentum since being founded in 2018 (1). The very first COIN ThinkTank brought together cardiologists, oncologists, and other specialists in August 2021 to facilitate meaningful discourse and information dissemination. ThinkTank agenda items were determined by network members at previous COIN meetings. The following topics were discussed by small working groups: AI and digital health in cardio-oncology (innovation); the role of informatics in G-COR (collaboration); education and innovative methods of education delivery in cardio-oncology (education). Cardiology, oncology, and industry leaders, as well as patient advocates, participated in the breakout sessions.

Cardiologists can collaborate with oncologists and radiologists to expedite the collection of imaging studies for cardio-oncology AI research. Recent reviews have cataloged various ways in which AI is being applied to cardiovascular imaging in cardio-oncology (5–8). Future app development may facilitate patient enrollment and engagement for prospective validation studies. Future AI and digital health innovations in cardio-oncology will also incorporate the SMART on FHIR architecture for app integration into electronic health records (1). The global cardio-oncology registry (G-COR) was recently established to examine disparities and variation in cardio-oncology care worldwide (1). COIN will collaborate on using informatics in the registry, especially to identify cancer survivors who are undertreated for their cardiovascular risk or disease.

In cardio-oncology, mapping the patient journey and touchpoints for disease or risk assessment and management could be beneficial. The patient journey can be determined in part from social listening to patients' needs and experiences on social media (9–11), and discussions with patient advocates (12). The patient journey map and other innovative methods of education can become instrumental in cardio-oncology. Additionally, the COIN Annual Summit in December each year (December 10^th^ in 2022; cardioonccoin.org) provides live continual professional development (12), with subsequent use of the summit presentation content as online enduring CME content *via* the COIN website. Within this, we share a draft of the map with colleagues and patient advocates, we can invite others to suggest additional touchpoints. On the COIN website, an education emphasis working group has been created to connect and collaborate on ideas such as the patient journey concept map. These discussions' outcomes can be shared on social media for others to view, discuss, and generate new ideas.

The ThinkTank examined next steps for enhancing cardiologists' and oncologists' collaboration, such as in G-COR, to address health disparities. Eliminating barriers to care is essential for achieving racial and ethnic health equity. In G-COR, ethnicity, income, insurance, transportation, and internet access, as well as the impact of socioeconomic status, ethnicity, and location on access to medical care will be investigated with proposal and testing of potential solutions. This will facilitate taking next steps in the pursuit of health equity in cardio-oncology (1, 13–17).

Cardio-oncology is a relatively new subspecialty of cardiology. The very first COIN ThinkTank brought together cardiologists, oncologists, and other specialists, along with patient advocates in August 2021. In small working groups, AI and digital health in cardio-oncology (innovation); the role of informatics in G-COR; and innovation education/innovative education methods were discussed. Emphasis was placed on recruiting more oncologists, addressing health equity, and advancing prevention in cardio-oncology *via* innovation and collaboration. Mapping the patient journey, engaging patients and social media, and appreciating resilience were all topics of interest. Together, in the face of all obstacles alongside our patients who are all very brave (4, 18–20), we can continue to motivate and inspire each in all that we do in the Cardiology Oncology Innovation Network.

All are welcome to join us at the next COIN ThinkTank on August 7, 2022.

## Disclosure

Our authors work closely with several health technology companies, none of whom inappropriately restrict or limit our analyses or publications. Industry and pharmaceutical companies sponsored the inaugural COIN ThinkTank 2021. Our sponsors did not exert any restrictions on our discussion, work, or publications.

## Author contributions

Conception and design: S-AB. All authors contributed to drafting of the manuscript, interpretation of data, critical revision, and final approval of manuscript.

## Funding

The authors declare that this study received funding from the National Center for Advancing Translational Sciences, National Institutes of Health. The funder was not involved in the study design, collection, analysis, interpretation of data, the writing of this article or the decision to submit it for publication.

## Conflict of interest

The authors declare that the research was conducted in the absence of any commercial or financial relationships that could be construed as a potential conflict of interest.

## Publisher's note

All claims expressed in this article are solely those of the authors and do not necessarily represent those of their affiliated organizations, or those of the publisher, the editors and the reviewers. Any product that may be evaluated in this article, or claim that may be made by its manufacturer, is not guaranteed or endorsed by the publisher.

## Author disclaimer

The contents are solely the responsibility of the authors and do not necessarily represent the official views of the NIH.
